# A Case of Spermatocytic Tumor of Testis

**DOI:** 10.1002/iju5.70089

**Published:** 2025-10-22

**Authors:** Satsuki Nagamine, Yosuke Nakanishi, Kei Tanaka, Asuka Ashikari, Ryu Kimura, Shotaro Nakanishi, Rin Kawamitsu, Naoki Wada, Akihiro Nishie, Junichi Inokuchi

**Affiliations:** ^1^ Department of Urology, Graduate School of Medicine University of the Ryukyus Ginowan Japan; ^2^ Department of Diagnostic Pathology University of the Ryukyus Hospital Ginowan Japan; ^3^ Department of Pathology and Oncology, Graduate School of Medicine University of the Ryukyus Ginowan Japan; ^4^ Department of Radiology, Graduate School of Medicine University of the Ryukyus Ginowan Japan

**Keywords:** non‐germ cell neoplasia in situ, seprmatocytic seminoma, spermatocytic tumor, testicular cancer, testicular germ cell tumor

## Abstract

**Background:**

Spermatocytic tumor is a rare type of testicular germ cell tumor, accounting for approximately 1% of all testicular neoplasms.

**Case Presentation:**

A 51‐year‐old male presented to the hospital with a painless mass in the left testis. Ultrasonography revealed a heterogeneous intratesticular mass with cystic components. Testicular tumor markers were within normal ranges. The patient underwent a left orchiectomy. Pathologically, the tumor was characterized by the presence of medium to large neoplastic cells and small neoplastic cells without sarcomatoid or anaplastic features. Immunohistochemically, the tumor was positive for SALL4 and negative for CD30, AFP, OCT3/4, PLAP, D2‐40, and hCG. Based on these findings, we diagnosed spermatocytic tumor of testis.

**Conclusion:**

We present a case of spermatocytic tumor, which is a distinct entity among testicular germ cell tumors, with a generally favorable prognosis following orchiectomy. However, long‐term follow‐up is recommended due to the potential for late metastasis.

**Summary**

- Spermatocytic tumor is a rare type of testicular germ cell tumor, accounting for approximately 1% of all testicular neoplasms, and is more prevalent in patients aged 50 and older.
- Although most patients with spermatocytic tumor can be cured by orchiectomy, those with sarcomatoid or anaplastic subtypes have a higher risk of recurrence.

## 1 Introduction

Spermatocytic tumor is an extremely rare type of testicular germ cell tumor (GCT), accounting for approximately 1% of all testicular tumors [1]. Testicular tumors are classified as GCT or non‐GCT, with more than 95% originating from germ cell lineage. Spermatocytic tumor was considered closely related to seminoma and classified as a subtype of this disease, referred to as spermatocytic seminoma, until the revision in the fourth edition of the World Health Organization (WHO) classification of urogenital tumors published in 2016 [2]. Spermatocytic tumors are not derived from germ cell neoplasia in situ (GCNIS) and lack chromosome 12p alterations [3], and show a unique amplification of Chromosome 9 corresponding to the *DMRT1* gene and are never associated with other forms of GCTs [4]. Spermatocytic tumor has different clinical and pathological features compared with other GCTs and is currently classified within the non‐GCNIS [5]. Here, we present a case of spermatocytic tumor of testis.

## 2 Case Presentation

A 51‐year‐old male was aware of a painless mass with induration in the left scrotum. However, he did not go to the hospital because he felt it shrank after that. One year later, the mass had grown again without pain. He went to the local clinic and was diagnosed with suspected testicular cancer. He was then referred to our hospital for consultation.

**FIGURE 1:**
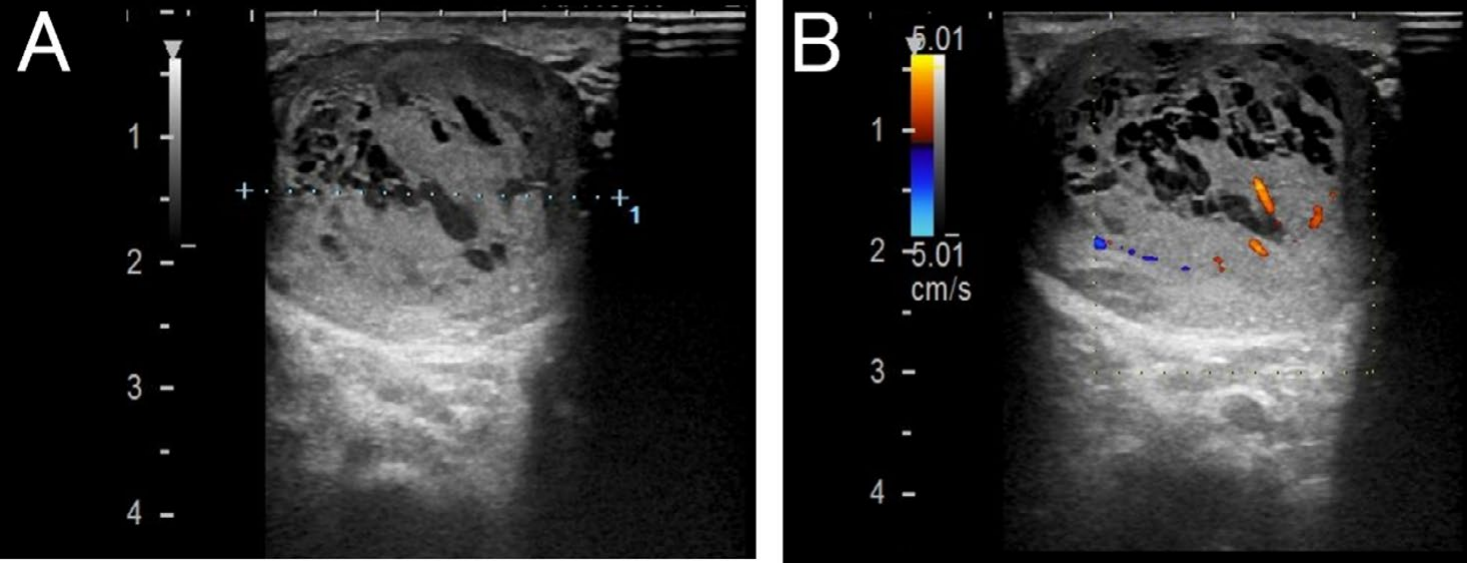
Ultrasonographic findings of the left testis. (A) The tumor occupies most of the left testis measuring 3 × 4.4 × 2.6 cm in size. Tumor showed heterogeneous echogenicity, and has some anechoic cystic lesions. (B) Color Doppler showed blood flow inside the tumor.

**FIGURE 2:**
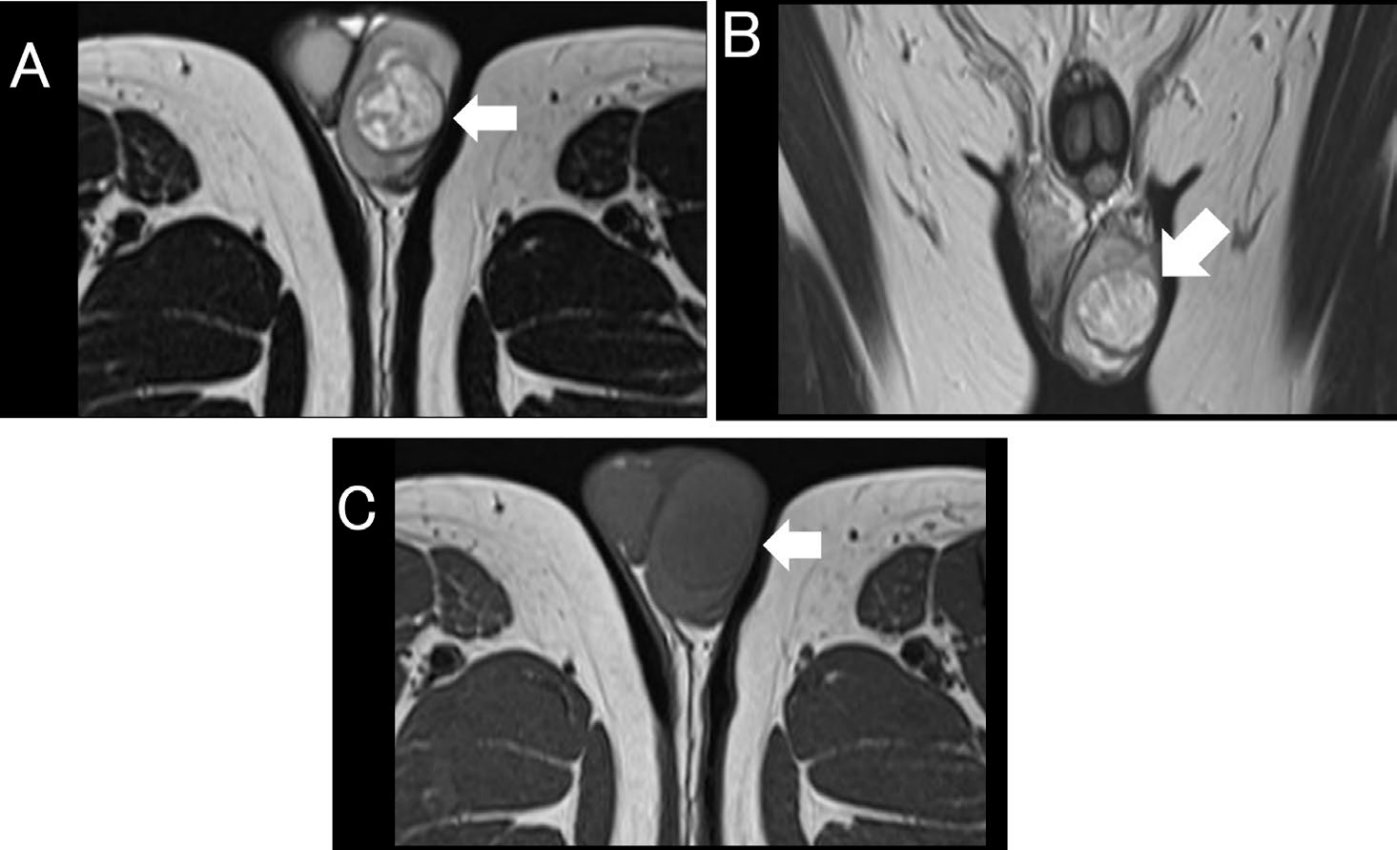
MRI findings of the left testis. (A, B) MRI showed tumor with a heterogenous high‐signal intensity and low‐signal capsule on the axial and coronal T2‐weighted images. (C) The tumor showed a uniform low‐signal intensity on the T1‐weighted MRI axial image.

At the time of referral, physical examination revealed a painless induration of the left scrotum, but no palpable enlarged inguinal lymph nodes. He had normal laboratory values, including normal tumor markers for testicular cancer, alpha‐fetoprotein (AFP), human chorionic gonadotropin (hCG), and lactate dehydrogenase (LDH). Ultrasonography revealed the presence of a mass occupying most of the left testis, showing a mixture of hypoechoic and isoechoic areas. Doppler ultrasonography indicated blood flow inside the tumor. In addition, the tumor had some anechoic cystic components (Figure 1). The magnetic resonance imaging (MRI) showed the tumor depicted as a uniform low‐signal area on the T1‐weighted images and as a well‐defined mass with a heterogeneous high‐signal intensity and low‐signal capsule on the T2‐weighted images (Figure 2). Contrast‐enhanced computed tomography (CT) of the thorax and abdomen showed no evidence of lymph node or distant metastasis. Due to the suspicion of testicular cancer, the patient underwent left orchidectomy. The left testis with the tumor was removed without any adhesions to the surrounding tissue. He had no severe complications during the perioperative period and was discharged on the second postoperative day.

**FIGURE 3:**
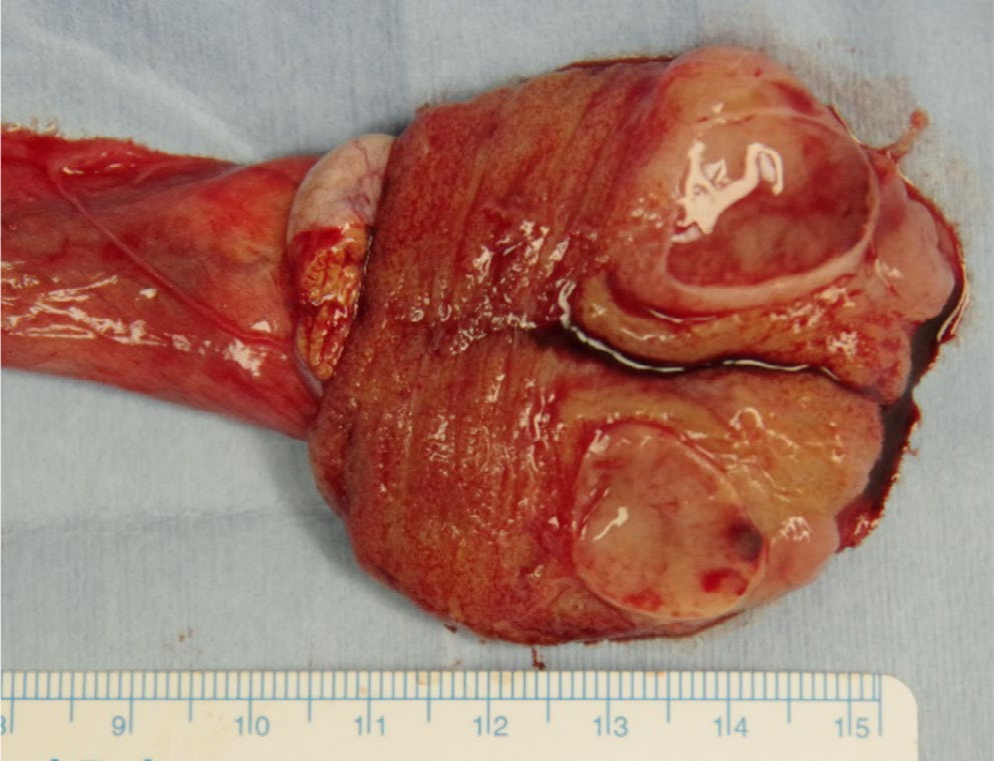
Marcoscopic finding of the removed testicular specimen.

**FIGURE 4:**
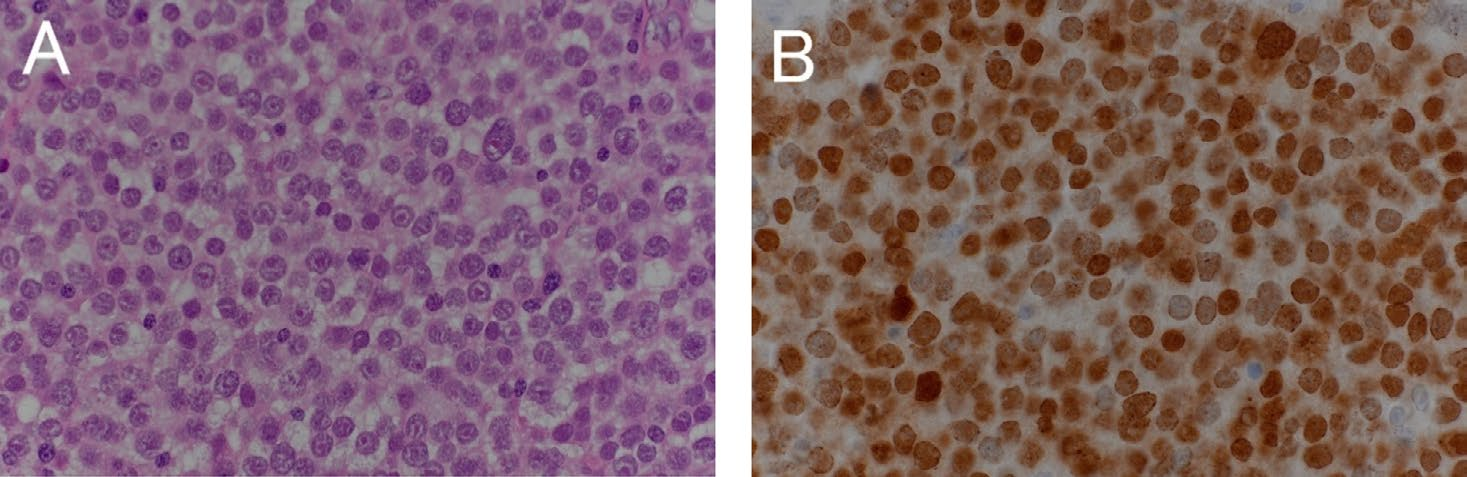
Immunohistochemical findings (A) Hematoxylin and eosin stained sections of the tumor composed of medium to large neoplastic cells and small neoplastic cells. (B) Positive immunohistochemical staining for SALL4.

Macroscopically, the tumor measured 15 × 13 × 25 mm and consisted of cystic structures and soft, pale yellow solid components (Figure 3). The histopathological examination showed the dense proliferation of medium to large neoplastic cells and small neoplastic cells. The tumor showed no sarcomatoid or anaplastic changes. Immunostaining examination showed positive staining for Sal‐like protein 4 (SALL4) (Figure 4) and negative staining for CD30, AFP, OCT3/4, placental alkaline phosphatase (PLAP), D2‐40, human chorionic gonadotropin (hCG) and leukocyte common antigen (LCA). Based on these results, we finally diagnosed the case as a spermatocytic tumor of thetestis.

Patients have been scheduled for a thoracoabdominal CT scan every 3 months for 2 years for postoperative recurrence and metastasis evaluation.

## 3 Discussion

Here we report a case of spermatocytic tumor of testis, which is an extremely rare histology among GCTs. In the recent analysis using the National Cancer Database, of 53 481 adults who received orchiectomy, 54.6% of patients were diagnosed with seminoma and only 0.6% with spermatocytic tumor [1]. In this cohort, patients with spermatocytic tumor were more likely to be older; the median age at diagnosis was 56.6 years compared with 38.5 years in seminoma [1]. These findings are consistent with previous studies; Hu et al. reported that 30% were from 30 to 39 years, 35% were from 40 to 49 years, and 35% were 60 years or older [6]. In men aged 50 years and older, the prevalence of testicular tumor types was reported as follows: 55.9% seminoma, 13.3% non‐seminoma, 25.9% malignant lymphoma, 3.2% spermatocytic tumor, and 1.7% sex cord stromal tumors [7].

Unilateral orchiectomy is the standard method for diagnosis and treatment; actually, more than 95% of patients were received without frozen section analysis [8]. Interestingly, several previous case reports showed ultrasonography images of this tumor, tumors with heterogeneous echogenicity and several small anechoic cystic lesions, similar to our case [9, 10]. These anechoic lesions displayed Doppler signal, indicating blood flow inside the tumors. These findings might be unique for spermatocytic tumor.

Histologically, spermatocytic tumor is a GCT derived from postpubertal‐type germ cells and consists of three types of cells: small cells with scant cytoplasm; intermediate cells with round nuclei; and large cells. Unlike GCTs, there is no GCNIS, and no significant inflammatory infiltrate, granulomas, fibrovascular septa, or cytoplasmic glycogen can be observed [5]. Immunohistochemically, spermatocytic tumors are negative for many GCT markers, including CD30, AFP, OCT3/4, PLAP, D2‐40, and hCG; they are positive for SALL4 [5].

Clinically, most spermatocytic tumors are considered to have a favorable prognosis. In a large retrospective study of testicular cancer patients, spermatocytic tumors showed approximately 80% of localized disease, 3.7% of regional nodal involvement, and only 0.7% of metastatic disease at diagnosis [1]. However, a systematic review indicated that metastases in patients with initially localized disease were diagnosed in 7% of patients and detected after a median follow‐up of 5.5 months (range 2–21 months), and the metastatic relapse was at the retroperitoneal lymph nodes, lung, liver, and brain [8]. Several reports indicated the risk factors for malignant behavior, including the sarcomatoid and anaplastic subtypes. The sarcomatoid subtype occurs in 6%–10% of spermatocytic tumors, while the anaplastic subtype occurs in 5% [8, 11]. The anaplastic subtype is rather monomorphic, with a uniform population of intermediate‐sized cells, in contrast to pure spermatocytic tumors [8, 12]. The systematic review revealed that 47% and 29% of patients with sarcomatoid or anaplastic subtypes had metastatic disease, respectively. Furthermore, patients with these histologic variants were more likely to have metastatic disease than those with pure spermatocytic tumors (41% vs. 5%) [8]. Recent genomic analyses revealed that malignant behavior was associated with relative gains of 12p and TP53 mutations [13]. Due to the rarity of this pathology, there is a paucity of data on the optimal management of patients with localized or metastatic disease and postoperative follow‐up. In a systematic review, only 4% of patients received adjuvant therapy, including platinum‐based chemotherapy, radiotherapy, and both [8]. In our case, because there was no sarcomatoid or anaplastic variant, no vascular invasion, no elevated tumor markers, and no evidence of distant metastasis, adjuvant therapy was not administered. We scheduled CT scans every 3 months for the first 2 years. Further accumulation of cases is needed to further clarify the pathogenesis of this disease.

## 4 Conclusion

Spermatocytic tumors are rare testicular tumors that develop in the 50s. Most patients with this tumor can be cured by orchiectomy. However, those with sarcomatoid or anaplastic subtypes have a higher risk of recurrence; therefore, long‐term follow‐up is recommended.

## Consent

The authors have nothing to report.

## Conflicts of Interest

The authors declare no conflicts of interest.

## References

[1] P. M. Patel , H. D. Patel , E. L. Koehne , et al., “Contemporary Trends in Presentation and Management of Spermatocytic Seminoma,” Urology 146 (2020): 177–182. 33049234 10.1016/j.urology.2020.10.002

[2] H. Moch , A. L. Cubilla , P. A. Humphrey , V. E. Reuter , and T. M. Ulbright , “The 2016 WHO Classification of Tumours of the Urinary System and Male Genital Organs‐Part A: Renal, Penile, and Testicular Tumours,” European Urology 70, no. 1 (2016): 93–105. 26935559 10.1016/j.eururo.2016.02.029

[3] C. Rosenberg , M. C. Mostert , T. B. Schut , et al., “Chromosomal Constitution of Human Spermatocytic Seminomas: Comparative Genomic Hybridization Supported by Conventional and Interphase Cytogenetics,” Genes, Chromosomes & Cancer 23, no. 4 (1998): 286–291. 9824200

[4] L. H. Looijenga , R. Hersmus , A. J. Gillis , et al., “Genomic and Expression Profiling of Human Spermatocytic Seminomas: Primary Spermatocyte as Tumorigenic Precursor and DMRT1 as Candidate Chromosome 9 Gene,” Cancer Research 66, no. 1 (2006): 290–302. 16397242 10.1158/0008-5472.CAN-05-2936

[5] S. Secondino , A. Viglio , G. Neri , et al., “Spermatocytic Tumor: A Review,” International Journal of Molecular Sciences 24, no. 11 (2023): 9529. 37298487 10.3390/ijms24119529 PMC10253486

[6] R. Hu , T. M. Ulbright , and R. H. Young , “Spermatocytic Seminoma: A Report of 85 Cases Emphasizing Its Morphologic Spectrum Including Some Aspects Not Widely Known,” American Journal of Surgical Pathology 43, no. 1 (2019): 1–11. 29280854 10.1097/PAS.0000000000001001

[7] A. A. Ghazarian , C. Rusner , B. Trabert , M. Braunlin , K. A. McGlynn , and A. Stang , “Testicular Cancer Among US Men Aged 50 Years and Older,” Cancer Epidemiology 55 (2018): 68–72. 29807233 10.1016/j.canep.2018.05.007 PMC6668029

[8] J. B. Grogg , K. Schneider , P. K. Bode , et al., “A Systematic Review of Treatment Outcomes in Localised and Metastatic Spermatocytic Tumors of the Testis,” Journal of Cancer Research and Clinical Oncology 145, no. 12 (2019): 3037–3045. 31646373 10.1007/s00432-019-03056-1 PMC11810242

[9] R. Bapir , I. Aghaways , R. M. Ali , et al., “Spermatocytic Tumor of the Testis: A Case Report and Mini‐Review of the Literature,” Medicine International 3, no. 5 (2023): 51. 37810904 10.3892/mi.2023.111 PMC10557091

[10] C. L. Petersen , P. O. Otto , S. Kjaer‐Frifeldt , and M. R. V. Pedersen , “Spermatocytic Tumors in 2 Patients Aged 50 and 77 Years: 2 Case Reports and Brief Review of the Literature,” Radiology Case Report 18, no. 10 (2023): 3572–3576. 10.1016/j.radcr.2023.07.043 PMC10412717 37577074

[11] A. F. Dias , E. Dvindenko , F. Santos , and R. Cabrera , “Sarcomatoid Spermatocytic Tumour: Report of a Rare Case and Literature Review,” International Journal of Surgical Pathology 31, no. 5 (2023): 728–733. 36128783 10.1177/10668969221122995 PMC10316531

[12] G. Mikuz , G. W. Bohm , M. Behrend , G. Schafer , M. Colecchia , and I. Verdorfer , “Therapy‐Resistant Metastasizing Anaplastic Spermatocytic Seminoma: A Cytogenetic Hybrid: A Case Report,” Analytical and Quantitative Cytopathology and Histopathology 36, no. 3 (2014): 177–182. 25141494

[13] S. Gupta , L. M. Sholl , Y. Yang , et al., “Genomic Analysis of Spermatocytic Tumors Demonstrates Recurrent Molecular Alterations in Cases With Malignant Clinical Behavior,” Journal of Pathology 262, no. 1 (2024): 50–60. 37792634 10.1002/path.6210

